# Analytical and Clinical Evaluation of the STANDARD M10 Arbovirus Panel for Dengue Detection, Serotyping, and Multiplex Arboviral Screening in the Americas

**DOI:** 10.3390/diagnostics16121799

**Published:** 2026-06-11

**Authors:** Stephany Young Yusty, Maria Chen-Germán, Dimelza Arauz, Melanie Vega, Lisseth Saenz, Mabel Martínez-Montero, Carlos Yanguez, Brechla Moreno, Gilberto A. Eskildsen

**Affiliations:** 1 Departamento de Microbiología Humana, Facultad de Medicina, Universidad de Panamá, Panama City 0816-00588, Panama; stephany.young@up.ac.pa; 2 Laboratorio Modular Especializado, Departamento de Virología y Biotecnología, Instituto Conmemorativo Gorgas para Estudios de la Salud, Panama City 0816-02593, Panama; mchen@gorgas.gob.pa (M.C.-G.);; 3 Sección de Inmunovirología del LCRSP, Instituto Conmemorativo Gorgas para Estudios de la Salud, Panama City 0816-02593, Panama; 4 Región Metropolitana de Salud, Ministerio de Salud de Panamá, Panama City 0816-02593, Panama

**Keywords:** arboviruses, Arbovirus surveillance, dengue (DENV), Zika (ZIKV), chikungunya (CHIKV), yellow fever (YFV), West Nile virus (WNV), molecular diagnostics, point-of-care testing (POCT)

## Abstract

**Background/Objectives:** Arboviruses including dengue virus (DENV), Zika virus (ZIKV), chikungunya virus (CHIKV), yellow fever virus (YFV), and West Nile virus (WNV) co-circulate across the Americas, generating overlapping febrile syndromes that challenge etiological diagnosis based solely on clinical criteria. Cartridge-based multiplex molecular platforms offer potential for decentralized testing in hyperendemic settings, yet independent real-world evaluations of their clinical and analytical performance remain limited. **Methods:** A retrospective two-phase analytical study was conducted. Phase 1 assessed clinical diagnostic accuracy for dengue using 163 de-identified serum samples classified using a composite reference standard consisting of Panbio NS1 ELISA reactivity (≥11 Panbio units) combined with compatible clinical and epidemiological data, operationalized in accordance with the PAHO 2023 laboratory confirmation algorithm for dengue; RT-qPCR was not routinely available for all archived samples, and reported sensitivity should therefore be interpreted as a conservative lower-bound estimate; Phase 2 evaluated analytical sensitivity across all eight panel targets using characterized arboviral reference strains in serial dilution experiments, with reference RT-qPCR assays as the comparator; this phase was incorporated to characterize detection thresholds for targets not represented by clinical specimens. **Results:** In Phase 1, the M10 demonstrated sensitivity of 96.0% (96/100), specificity of 100% (63/63), overall accuracy of 97.5%, and near-perfect agreement with the reference standard (Cohen’s κ = 0.95). DENV-3 was the predominant serotype (74/96; 77.1%), followed by DENV-1 (16.7%) and DENV-4 (6.3%); DENV-2 was not detected. In Phase 2, operational LoDs (defined as the lowest concentration yielding a detectable Ct in all triplicate reactions for the RT-qPCR, and from a single cartridge per dilution point for the STANDARD M10) were equivalent or superior to reference RT-qPCR for six targets (DENV-1, DENV-3, DENV-4, ZIKV, WNV, YFV; range 1–5 PFU/mL), while DENV-2 and CHIKV showed 20-fold higher operational LoDs (20 PFU/mL vs. 1 PFU/mL for the reference RT-qPCR); formal LoD_95_ estimates were not determined. **Conclusions:** The STANDARD M10 Arbovirus Panel shows high clinical accuracy for dengue and adequate analytical sensitivity for most targets, supporting its use as a complementary decentralized molecular tool. Reduced sensitivity for DENV-2 and CHIKV and the absence of formal LoD_95_ estimates remain key limitations to be addressed in future validation studies.

## 1. Introduction

Arboviral diseases represent a major and growing global public health threat, with a disproportionate burden in tropical and subtropical regions driven by complex ecological, environmental, and epidemiological factors that promote viral emergence and re-emergence [1,2,3,4]. Among the most medically important arboviruses are members of the families *Flaviviridae* and *Togaviridae* [5]. The former includes Dengue virus (DENV), Zika virus (ZIKV), Yellow Fever virus (YFV), and West Nile virus (WNV), whereas Chikungunya virus (CHIKV) belongs to the family *Togaviridae* [6]. In recent years, these viruses have undergone notable shifts in their epidemiology, geographic distribution, and transmission dynamics, with significant public health impact across the Americas and beyond [7,8].

Dengue virus, the most widespread human arbovirus, comprises four antigenically distinct serotypes (DENV-1–4) and has undergone the most dramatic epidemiological expansion in recent decades [3,9,10,11]. In 2024, WHO and PAHO reported nearly 14 million dengue cases globally, of which approximately 13 million were recorded in the Region of the Americas, accompanied by co-circulation of all four serotypes in multiple countries [3,12]. Although dengue transmission in the Americas declined by approximately two-thirds in 2025 relative to the 2024 peak, the persistently elevated arboviral burden continues to impose substantial diagnostic challenges on regional health systems [3,11,13,14,15].

Similarly, CHIKV has re-emerged in several countries following years of relatively limited activity [16]. Major outbreaks have been documented in Brazil, Bolivia, Paraguay, and Cuba, underscoring the capacity of CHIKV to rapidly re-establish transmission in both endemic and previously affected regions [17,18,19]. Concurrently, YFV has shown a marked increase in incidence and geographic expansion [2]: during 2025, reported cases increased more than fivefold relative to the preceding year, with confirmed transmission in Colombia, Venezuela, Bolivia, and Brazil [20].

Although ZIKV and WNV infections are frequently asymptomatic or self-limiting, both can cause severe clinical outcomes and represent ongoing public health concerns [21,22]. ZIKV is of particular importance because of its association with congenital abnormalities, including congenital Zika syndrome, following infection during pregnancy [23]; since the 2015–2016 epidemic in the Americas, transmission has persisted at low levels, with ongoing circulation documented primarily in Brazil [24]. WNV is a leading cause of neuroinvasive arboviral disease, producing meningitis, encephalitis, acute flaccid paralysis, and long-term neurological sequelae; although reported predominantly in the United States, human and animal infections have been documented in Brazil and other countries, reinforcing the need for vigilant surveillance and robust diagnostic capacity across the region [21,25,26,27].

The co-circulation of these arboviruses generates overlapping acute febrile syndromes that preclude etiological differentiation based solely on clinical criteria [9,10,12,28,29]. Co-infections involving distinct DENV serotypes and combinations such as DENV–ZIKV and DENV–CHIKV have been documented across Latin America, Asia, and Africa; sequential heterologous DENV infections are associated with antibody-dependent enhancement and increased severity risk, underscoring the diagnostic importance of serotype-level resolution [16,30,31].

Laboratory diagnosis of arboviruses relies on the detection of viral nucleic acids, NS1 antigen, or virus-specific antibodies, with test selection determined by the timing of specimen collection relative to symptom onset [32]. Since the emergence of ZIKV in the Americas in 2015, real-time RT-qPCR has become the preferred method for acute-phase arboviral diagnosis, offering superior specificity, improved early-infection sensitivity, etiological discrimination among co-circulating viruses, and reduced susceptibility to cross-reactivity within the family Flaviviridae [11,32,33,34]. Serological methods retain an important complementary role in post-viremic diagnosis and epidemiological surveillance [35,36].

The co-circulation of multiple arboviruses has driven the development of multiplex RT-qPCR assays capable of simultaneously detecting several viral targets, including all four DENV serotypes, in a single reaction [34,37,38,39,40]. Several of these assays have demonstrated high analytical performance for DENV and CHIKV, improved diagnostic accuracy relative to NS1/IgM ELISA, and the capacity to identify co-infections in high-transmission settings [33,37,39,41]. Most multiplex molecular systems, however, require specialized laboratory infrastructure, separate nucleic acid extraction procedures, trained personnel, and controlled cold-chain storage [42]. Closed-cartridge sample-to-answer platforms address these operational constraints by integrating extraction, amplification, and detection into a single automated workflow, reducing hands-on time, contamination risk, and infrastructure requirements while enabling deployment closer to the point of care [42].

In Panama, a country with hyperendemic dengue transmission and documented circulation of ZIKV, CHIKV, and other arboviruses, diagnostic capacity varies considerably across health facilities [43]. Although the national reference laboratory and a limited number of specialized centers have access to molecular platforms, many facilities face constraints in infrastructure, equipment, and trained personnel that restrict routine implementation of conventional RT-qPCR assays [1,44,45,46], potentially delaying etiological confirmation and timely case reporting. In this context, independent evaluation of cartridge-based multiplex platforms under real-world conditions is particularly relevant for benchmarking performance against established reference assays and assessing their capacity to support decentralized arboviral detection [34,39,45,46,47,48,49,50]. The STANDARD M10 Arbovirus Panel (SD Biosensor) is a fully automated, closed-cartridge assay that simultaneously detects DENV-1–4, ZIKV, CHIKV, YFV, and WNV directly from serum or plasma within approximately 60 min [51]. Independent peer-reviewed evaluations of this platform under routine diagnostic conditions in hyperendemic settings in the Americas remain scarce, representing a meaningful evidence gap for implementation decisions in public health and decentralized laboratories.

To address this gap, this study evaluated the clinical diagnostic accuracy and analytical sensitivity of the STANDARD M10 Arbovirus Panel using a two-phase design. Phase 1 assessed real-world clinical performance for dengue detection and serotyping using acute-phase serum samples from a hyperendemic setting in Panama. Phase 2 characterized the analytical performance of the panel across all eight targets using characterized reference strains under controlled conditions; this phase was incorporated to address the unavailability of confirmed clinical specimens for ZIKV, CHIKV, YFV, and WNV in the study setting, and provides detection threshold data relevant for assessing the panel’s potential contribution to multiplex arboviral surveillance.

## 2. Materials and Methods

### 2.1. Study Design and Setting

A retrospective analytical study was conducted in two complementary phases. Phase 1 assessed the clinical diagnostic performance of the STANDARD M10 Arbovirus panel (SD Biosensor; Seoul, Republic of Korea) using serum samples from patients with suspected acute dengue (≤5 days of symptoms) and previously characterized archived sera classified as reference-positive by NS1 ELISA plus compatible clinical–epidemiological data. Phase 2 evaluated the analytical sensitivity and cycle threshold (Ct) profiles of the STANDARD M10 across all eight panel targets in comparison with reference RT-qPCR assays, using characterized arboviral strains at titrated concentrations. This phase was designed to provide analytical performance data for targets that could not be assessed in the clinical phase due to the unavailability of confirmed clinical specimens for ZIKV, CHIKV, YFV, and WNV in the study setting. The study was carried out at the Department of Human Microbiology of the Universidad de Panama, in collaboration with the Specialized Modular Virology Laboratory and the Immunovirology Section of the Gorgas Memorial Institute for Health Studies (ICGES) in Panama City, Panama. This study was reported in accordance with the Standards for Reporting of Diagnostic Accuracy Studies (STARD) 2015 guidelines [52]. The completed STARD 2015 checklist is provided as Supplementary Table S3.

### 2.2. Phase 1: Clinical Diagnostic Performance

A total of 163 de-identified serum samples were included, comprising 101 samples obtained in 2025 from symptomatic patients with suspected acute dengue attending health centers and laboratories of the Metropolitan Health Region of the Ministry of Health of Panama, and 62 archived samples from the Immunovirology Section of the Gorgas Memorial Institute for Health Studies (ICGES) collected through an external quality assessment program. Archived samples from ICGES had been stored at −80 °C with no more than 1 freeze–thaw cycle. Storage conditions were verified prior to inclusion. Samples were selected by convenience sampling from those fulfilling the predefined inclusion criteria: patients with clinical suspicion of arboviral infection (e.g., dengue, Zika, chikungunya), presentation in the acute phase (0–5 days from symptom onset), venous blood collected in a sterile tube without anticoagulant, and availability of the corresponding clinical data collection form.

Exclusion criteria comprised absence of the completed form, serum volume <1 mL, lipemic or hemolyzed specimens, and convalescent-phase samples with more than 5 days since symptom onset. Of 200 initially screened sera, 37 were excluded due to insufficient volume, incomplete documentation, or poor sample quality, yielding a final analytical dataset of 163 samples. All included sera had been previously processed using NS1 ELISA assays and were classified as acute according to clinical information and time since symptom onset. No formal prospective sample size calculation was performed; the study included all archived specimens meeting inclusion criteria available at the time of the evaluation. Post hoc power considerations indicate that estimating sensitivity of ≥90% with a margin of error of ±5% at 95% CI requires a minimum of approximately 97 positive samples using exact binomial methods; the inclusion of 100 reference-positive samples therefore provides sufficient precision to estimate sensitivity within clinically meaningful bounds, and the 63 reference-negative samples provide adequate precision for specificity estimation.

Dengue case classification in Phase 1 was based on a composite reference standard integrating NS1 antigen reactivity by Panbio Dengue Early ELISA with compatible clinical and epidemiological information. This approach aligns with the PAHO 2023 laboratory confirmation algorithm, which explicitly positions NS1 ELISA as a virological confirmatory test for dengue in acute-phase samples [1,44], and is consistent with national diagnostic practice at ICGES and MINSA. The composite design was selected to maximize the specificity of case classification: samples required both NS1 ELISA positivity (≥11 Panbio units) and clinical–epidemiological compatibility to be classified as dengue-positive, thereby reducing misclassification of cross-reactive or non-dengue febrile illness [35,53]. This approach has precedent in the literature; composite NS1-based reference standards have been applied in diagnostic evaluations conducted in settings where real-time RT-qPCR was not universally available, and the Panbio Dengue Early ELISA has demonstrated high specificity (≥99%) for dengue confirmation in acute-phase samples across multiple independent evaluations [36,54].

Although RT-qPCR represents the preferred reference method when full molecular infrastructure is available, its routine application to archived samples from geographically distributed peripheral health centers was not operationally feasible, reflecting the diagnostic reality of most facilities outside national reference laboratories in Panama [55]. Critically, because NS1-negative, RT-qPCR-detectable dengue cases, which arise predominantly in secondary infections with lower viraemia, would be misclassified as reference-negative, the sensitivity of 96.0% reported here should be interpreted as a conservative lower-bound estimate of the M10’s true clinical sensitivity against an RT-qPCR gold standard.

Samples negative for dengue NS1 antigen by ELISA were classified as reference-negative. The Panbio Dengue Early ELISA (Abbott Laboratories, Chicago, IL, USA) was performed according to the manufacturer’s instructions. Optical densities were read at 450 nm (reference 600–650 nm) and results interpreted using predefined cut-offs: negative (<9 Panbio units), equivocal (9–11 units), and positive (>11 units) [36,56,57].

For testing with the STANDARD M10 Arbovirus Panel, 600 µL of serum was loaded into a single-use cartridge and processed according to the manufacturer’s instructions [51]. All assays were performed by trained personnel blinded to the composite reference classification at the time of testing. Reference standard classifications had been assigned prior to and independently of M10 testing, ensuring bidirectional blinding.

### 2.3. Phase 2: Analytical Sensitivity and Ct Profiles

#### 2.3.1. Viral Isolates and Cells

**Figure 1:**
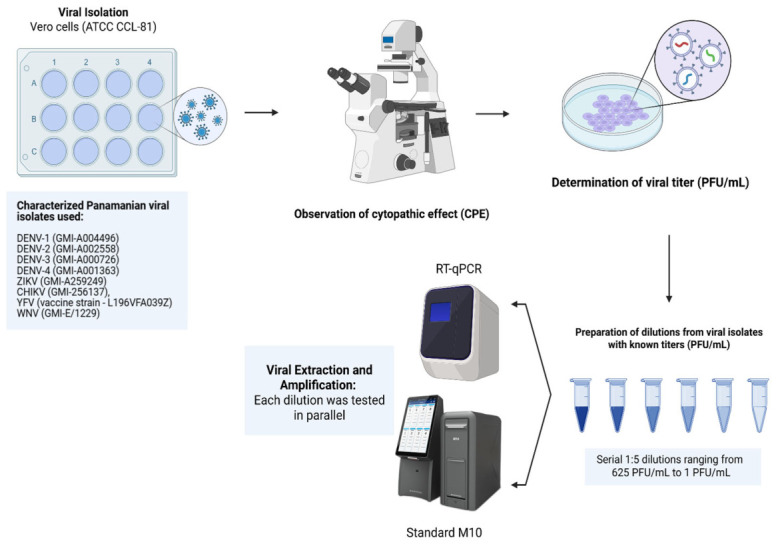
Experimental workflow for the analytical sensitivity evaluation of the STANDARD M10 Arbovirus Panel. Characterized viral isolates were propagated in Vero cells; viral stocks were harvested upon cytopathic effect and titrated by plaque assay. Fivefold (1:5) serial dilutions (625–1 PFU/mL) were prepared for each virus, with additional intermediate points for DENV-2, DENV-4, and CHIKV. Each dilution was tested in parallel: 600 µL was loaded directly into individual M10 cartridges (no prior extraction), and 200 µL underwent automated RNA extraction followed by reference RT-qPCR amplification in triplicate. Created in BioRender. Young, S. (2026) https://BioRender.com/oro6wzx (accessed on 21 April 2026).

Eight characterized Panamanian viral isolates were used: DENV-1 (GMI-A004496), DENV-2 (GMI-A002558), DENV-3 (GMI-A000726), DENV-4 (GMI-A001363), ZIKV (GMI-A259249), CHIKV (GMI-256137), YFV vaccine strain (L196VFA039Z), and WNV (GMI-E/1229), all obtained from the Virology Department repository of ICGES and previously characterized by molecular methods (Figure 1).

Vero cells (ATCC CCL-81) were used for the production of viral stocks. Cells were maintained at 37 °C and 5% CO_2_ in Minimum Essential Medium (MEM) supplemented with 2% fetal bovine serum, 1% penicillin–streptomycin, and 0.5% amphotericin B. Viruses were harvested after 3–7 days depending on the virus and titrated to determine their plaque-forming unit concentration (PFU/mL) as previously described [58]. Cytopathic effect (CPE) was monitored daily by inverted light microscopy as an indicator of productive viral replication (Figure 1). Observed changes included progressive cell rounding, cytoplasmic vacuolation, and monolayer detachment, with onset typically between days 3–5 for DENV serotypes and ZIKV, and within 48–72 h for CHIKV, which produced more rapid and pronounced CPE. YFV (vaccine strain 17D) and WNV produced moderate cytopathic changes with focal lysis, generally apparent between days 4–7. Cultures were harvested at 70–80% CPE to balance viral yield and RNA integrity.

#### 2.3.2. Dilutions, Viral Extraction and Amplification

Based on the known concentration of each virus (PFU/mL), serial 1:5 dilutions were prepared, ranging from 625 PFU/mL to 1 PFU/mL. For DENV-2, DENV-4, and CHIKV, additional intermediate dilution points (20 and 15 PFU/mL) were prepared to more precisely characterize the operational limit of detection in the region where signal loss was anticipated. Each dilution was tested in parallel: 600 µL was loaded directly into individual M10 cartridges without prior extraction, while 200 µL underwent automated RNA extraction using the MagMAX™ Viral/Pathogen II Nucleic Acid Isolation Kit (Thermo Fisher Scientific, Waltham, MA, USA, Cat. A48383) on the KingFisher Flex instrument, prior to RT-qPCR amplification in triplicate on an Applied Biosystems QuantStudio™ 5 Real-Time PCR System (Figure 1). Amplification was performed using individual protocols for each target [59,60,61,62] except for dengue virus, for which a single multiplex reaction was used to detect and differentiate all four serotypes. All reference RT-qPCR reactions were performed using the iTaq™ Universal Probes One-Step Kit (Cat. No. 1725141, Bio-Rad Laboratories; Berkeley, CA, USA); mean Ct values were calculated from the three replicates. For the STANDARD M10, each dilution point was tested using one cartridge, yielding a single Ct measurement per concentration, as the closed-cartridge platform does not permit within-run technical replication. Analytical sensitivity and Ct values obtained with the STANDARD M10 were compared with those generated by the reference RT-qPCR assays across all dilution points.

#### 2.3.3. Calibration Curve Construction and Limit of Detection Determination

The analytical sensitivity analyses were performed using the Ct values obtained from fivefold (1:5) serial dilutions of cultured virus, expressed in plaque-forming units per milliliter (PFU/mL), tested in parallel by the STANDARD M10 Arbovirus Panel and the reference RT-qPCR assays. For each virus, Ct values were recorded at each dilution point and organized in a structured dataset comprising virus, dilution (PFU/mL), log_10_-transformed concentration, mean Ct for the RT-qPCR (mean of triplicates), and single Ct for the STANDARD M10 (one cartridge per dilution point). Detailed Ct values are provided in Supplementary Table S2.

**Figure 2:**
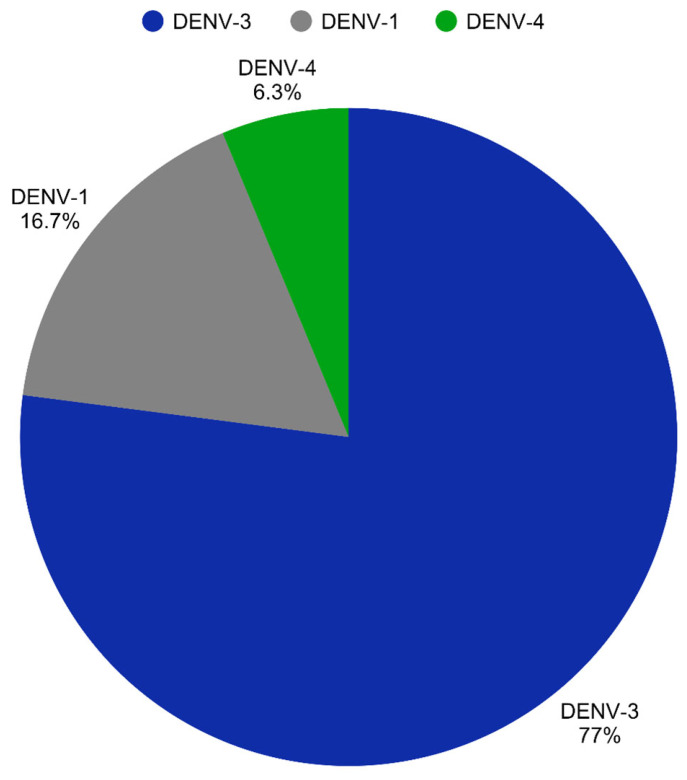
Serotype distribution among dengue-positive serum samples detected by the STANDARD M10 Arbovirus Panel. Proportional distribution of DENV serotypes among dengue reference-positive serum samples (Phase 1, January–June 2025), as determined by the STANDARD M10 target-specific channel results, which simultaneously identify DENV-1, DENV-2, DENV-3, and DENV-4 in a single reaction. DENV-3 was the predominant serotype, followed by DENV-1 and DENV-4. DENV-2 was not detected in any sample analyzed.

To generate the calibration curves (Figure 2), Ct values were modeled as a function of log10 PFU/mL using simple linear regression for each virus and method separately, according to the model Ct = β_0_ + β_1_ log_10_ (PFU/mL), where β_0_ represents the y-intercept and β_1_ the slope of the standard curve. Goodness of fit was assessed by visual inspection of residuals and by the coefficient of determination (R^2^). Scatter plots of Ct versus log10 PFU/mL were produced for each virus in a multi-panel faceted layout, overlaying the fitted regression lines for both methods to allow direct visual comparison of slope and intercept across analytes.

For each virus, the observed operational LoD was defined as the lowest concentration in the dilution series (PFU/mL) at which a Ct value was obtained: for the RT-qPCR, this required detection in all three triplicate reactions; for the STANDARD M10, detection was assessed from the single cartridge tested at each dilution point. These operational LoDs are descriptive in nature and do not correspond to LoD_95_ estimates derived from probit regression, which would require a minimum of 20 independent replicates at each concentration per CLSI EP17-A2 guidance and were outside the scope of this study. For the purpose of this analysis, a dilution was considered positive if the instrument generated a numeric Ct value; dilutions explicitly reported as ‘NEGATIVE’ (no amplification detected) were considered below the operational limit of detection. LoD values for both platforms are compared in Table 1 and detailed Ct values across the full dilution range are provided in Supplementary Table S2.

### 2.4. Statistical Analysis

In Phase 1, the sensitivity, specificity, and overall accuracy of the STANDARD M10 for dengue were calculated against the reference standard, with 95% confidence intervals (95% CI) estimated using exact binomial methods. In addition, an exploratory description of the DENV serotype distribution and the detection of other arboviruses within the cohort was provided.

In Phase 2, for each virus, exploratory linear regression models were fitted with Ct as the dependent variable and log_10_(PFU/mL), method (STANDARD M10 vs. RT qPCR) and their interaction as predictors, in order to characterize the relationship between viral concentration and Ct for both platforms. Ct values were modeled using the linear regression: Ct = β_0_ + β_1_log_10_(PFU/mL) + β_2_Method + β_3_log_10_(PFU/mL) × Method + ε where Method = 0 for the RT-qPCR and Method = 1 for the STANDARD M10 Arbovirus Panel. Regression coefficients and 95% confidence intervals based on a normal approximation are presented in Supplementary Table S4 and were interpreted as descriptive, given the limited number of dilution levels and the use of averaged Ct values per dilution.

Data management was performed in Python 3.0.1using the pandas library (v2.0) [63], and statistical modeling and graphical output were produced in R (version 4.1.0) [64]. Because both methods were evaluated across matched dilution points and the number of concentration levels per virus was limited (*n* = 5 for DENV-1, DENV-3, ZIKV, WNV, and YFV; *n* = 7 for DENV-2 and CHIKV, which included additional intermediate points; and DENV-4 was tested at *n* = 7 levels), the between-method Ct comparisons derived from these models are descriptive and exploratory in nature, and should not be interpreted as definitive inferential analyses.

## 3. Results

### 3.1. Phase 1: Clinical Diagnostic Performance

The STANDARD M10 identified 96 of 100 dengue reference-positive sera and 63 of 63 reference-negative sera, with no false positives. Sensitivity was 96.0% (95% CI: 89.8–98.9), specificity was 100% (95% CI: 94.3–100), overall accuracy was 97.5%, and Cohen’s κ was 0.95 (95% CI: 0.90–1.00), indicating near-perfect agreement (Table 1).

Among the dengue-positive samples identified during the first half of 2025 (January–June 2025), DENV-3 was the predominant serotype detected by the STANDARD M10 Arbovirus Panel, accounting for 74 of 96 (77.1%) positive detections. DENV-1 represented 16.7% of positive samples (16/96), while DENV-4 accounted for 6.3% (6/96). Notably, DENV-2 was not detected in any of the samples analyzed (Figure 2). This distribution aligns with PAHO surveillance alerts reporting the re-emergence of DENV-3 across the Americas [65].

Clinical specimens from patients with confirmed ZIKV, CHIKV, YFV, or WNV infection were not available in the study setting; accordingly, formal sensitivity and specificity estimate for these targets could not be derived from clinical data. The STANDARD M10 generated no discordant positive results among reference-negative samples, providing indirect evidence of specificity for non-dengue targets in the context of dengue-predominant acute febrile illness. To address the absence of confirmed clinical specimens for these pathogens, Phase 2 was designed to characterize the analytical detection limits of the panel across all eight targets under controlled conditions, providing a complementary evidence base for interpreting the platform’s potential for multiplex arboviral screening. Furthermore, analytical specificity was confirmed in controlled multiplex testing across eight viral combinations, including co-circulation scenarios; no cross-reactivity or misclassification was observed for any panel virus, and the non-panel virus Oropouche did not trigger false-positive signals on any channel (Supplementary Table S1).

### 3.2. Phase 2: Analytical Sensitivity and Cycle Threshold (Ct) Profiles

#### Observed Operational Limit of Detection: M10 vs. RT (PFU/mL)

The observed operational limit of detection (LoD) varied between platforms depending on the virus (Table 2). For DENV-1 and YFV, the M10 demonstrated superior sensitivity, with LoDs of 1 PFU/mL compared with 5 PFU/mL for the reference RT-qPCR. For DENV-3, ZIKV, WNV, and DENV-4, both platforms showed equivalent performance (LoD: 1, 1, 1, and 5 PFU/mL, respectively, for both methods). In contrast, the reference RT-qPCR demonstrated lower LoDs for DENV-2 (1 vs. 20 PFU/mL for M10) and CHIKV (1 vs. 20 PFU/mL for M10).

**Table 2:** Comparison of observed operational limits of detection (LoD) between the STANDARD M10 Arbovirus Panel and the reference RT-qPCR. For the RT-qPCR, the operational LoD was defined as the lowest concentration (PFU/mL) yielding a detectable Ct value in all three triplicate reactions; for the STANDARD M10, it was defined as the lowest concentration yielding a detectable Ct from the single cartridge tested per dilution point. Serial 1:5 dilutions (625–1 PFU/mL) were tested for each virus; additional intermediate points (20 and 15 PFU/mL) were included for DENV-2, DENV-4, and CHIKV. Full Ct values are provided in Supplementary Table S2.

	M10	RT-qPCR
DENV-1	1 PFU/mL	5 PFU/mL
DENV-2	20 PFU/mL	1 PFU/mL
DENV-3	1 PFU/mL	1 PFU/mL
DENV-4	5 PFU/mL	5 PFU/mL
ZIKV	1 PFU/mL	1 PFU/mL
CHIKV	20 PFU/mL	1 PFU/mL
WNV	1 PFU/mL	1 PFU/mL
YFV	1 PFU/mL	5 PFU/mL

Note: It should be noted that, given the single-cartridge design of M10 testing per dilution point, the operational LoDs reported for the M10 are based on single observations and may not capture run-to-run variability. The LoD values should therefore be regarded as indicative rather than definitive, and prospective replicated studies using formal probit analysis are warranted for regulatory-grade LoD_95_ determination.

### 3.3. Calibration Curves and Comparative Ct Profiles

**Figure 3:**
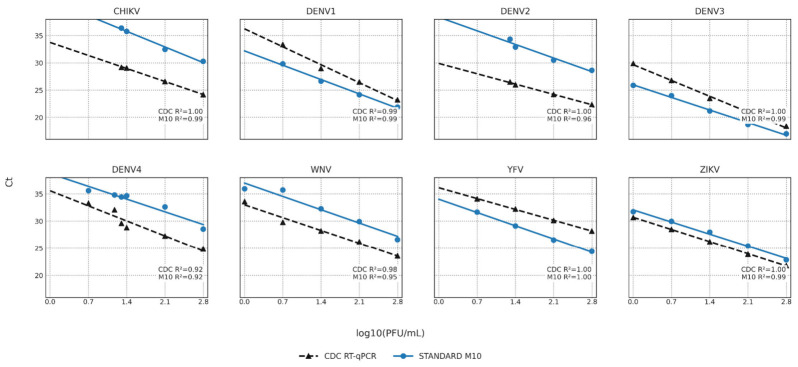
Comparative analytical performance and linear regression analysis of the STANDARD M10 Arbovirus Panel and RT-qPCR assays across a fivefold (1:5) serial dilution series (625–1 PFU/mL). The relationship between cycle threshold (Ct) values and viral concentration (log_10_ PFU/mL) is shown. Data points represent the mean Ct value of triplicate measurements for the RT-qPCR assay; for the STANDARD M10, each data point represents a single Ct measurement per cartridge, reflecting the closed-system design of the platform, which precludes within-run technical replication. Regression lines represent linear fits for the STANDARD M10 Arbovirus Panel (solid blue) and the RT-qPCR (dashed black). R^2^ values for each virus–method combination is annotated within each panel.

For ZIKV and WNV, both platforms produced regression lines with similar slopes and modest positive Ct shifts for the M10, indicating comparable amplification efficiency and equivalent or near-equivalent LoDs for these targets (Figure 3). For DENV-3, the M10 regression line was shifted downward relative to the RT-qPCR, reflecting superior amplification efficiency, though equivalent LoDs (1 PFU/mL) were achieved by both methods. For YFV, the M10 assay consistently yielded lower Ct values throughout the dilution series and maintained detection at 1 PFU/mL, a concentration at which the RT-qPCR assay failed to produce a positive result. For DENV-1, the M10 regression line was shifted downward (toward lower Ct values), reflecting higher amplification efficiency of the M10 for this target relative to the RT-qPCR; detection was preserved down to 1 PFU/mL, consistent with the LoD reported in Table 2. For DENV-4, both platforms showed comparable regression lines with equivalent LoDs of 5 PFU/mL, and neither method detected the target at 1 PFU/mL. In contrast, for DENV-2 and CHIKV, the M10 regression lines were shifted upward by approximately 6–7 Ct units compared with the RT-qPCR assay, indicating reduced amplification efficiency at all tested concentrations and resulting in loss of detection at low viral loads, concordant with the higher LoDs observed for these two targets (Figure 3).

Linear regression models showed strong linearity for both platforms across all viruses (R^2^ range: 0.92–1.00 for all virus–method combinations), confirming a consistent relationship between Ct and viral concentration throughout the tested dilution range (625–1 PFU/mL). Exploratory regression modeling indicated systematic Ct differences between platforms across all eight targets; however, given the limited number of dilution levels and the use of averaged Ct values per concentration point, these differences should be interpreted as descriptive rather than as statistically confirmed inferences. The largest positive Ct shifts for the M10 relative to the reference RT-qPCR were observed for DENV-2 (ΔCt = +6.85) and CHIKV (ΔCt = +6.47), consistent with the higher LoDs for these two targets. For DENV-4 (ΔCt = +4.12) and WNV (ΔCt = +3.82), the M10 exhibited higher mean Ct values across the dilution range, though equivalent LoDs were achieved (5 PFU/mL and 1 PFU/mL, respectively), indicating similar analytical sensitivity at the detection threshold despite reduced amplification efficiency at higher concentrations. For ZIKV (ΔCt = +1.38), Ct values and LoDs were equivalent between platforms. Note that the mean Ct shifts reported here reflect the arithmetic mean of per-dilution Ct differences between platforms at matched concentrations where both assays produced detectable signals, and differ slightly from the regression-based intercept offsets (β_2_ coefficients) presented in Supplementary Table S4, which represent the modeled vertical shift of the M10 regression line relative to the RT-qPCR across the full dilution range.

## 4. Discussion

The present study provides an independent two-phase evaluation of the STANDARD M10 Arbovirus Panel. These results support the M10 as a viable molecular option for acute dengue diagnosis in settings where simplified, closed-cartridge workflows and rapid turnaround represent operational priorities, provided that the limitations of the reference standard and the target-dependent analytical performance described below are taken into account. In Phase 1, the assay achieved 96.0% sensitivity, 100% specificity, and 97.5% overall accuracy (Cohen’s κ = 0.95) against the composite NS1-based reference standard, consistent with recent independent assessments reporting sensitivity of approximately 93% and specificity of 100% relative to conventional RT-qPCR [39]. The use of a composite NS1 ELISA-based reference standard, rather than RT-qPCR, introduces a differential verification bias that must be acknowledged: published meta-analyses report pooled NS1 ELISA sensitivities of approximately 67–82% across primary and secondary infections, with markedly lower values in secondary infections due to immune complex formation [36,54,56]. Accordingly, NS1-negative, RT-qPCR-detectable cases, predominantly those with secondary infection or late-acute presentation, would be misclassified as reference-negative, potentially registering as M10 false positives against this reference and simultaneously deflating apparent sensitivity. The reported 96.0% sensitivity should therefore be interpreted as a conservative lower bound, and the 100% specificity as measured against an imperfect reference rather than against RT-qPCR confirmation. Future evaluations should incorporate RT-qPCR as a co-reference standard to quantify the magnitude of this bias.

In Phase 2, analytical performance was target-dependent. The M10 demonstrated limits of detection comparable to the reference RT-qPCR for six of eight targets, consistent with published data for other multiplex arboviral platforms including the TaqMan Arbovirus Triplex [33,66,67,68,69]. Analytical specificity was confirmed across all eight viral combinations with no cross-reactivity, and the non-panel virus Oropouche generated no false-positive signals (Supplementary Table S1). Manufacturer validation data similarly report high specificity and no mutual interference under competitive testing conditions [49,51].

The regression-based Ct offsets provide additional insight into the analytical performance patterns suggested by the operational LoD data. For ZIKV and WNV, the small positive Ct shifts for the STANDARD M10 (approximately +1 to +4 cycles) with slopes similar to the RT-qPCR indicate slightly later detection but preserved linearity across the tested dilution range, an effect that is unlikely to be clinically meaningful in acute-phase specimens. In contrast, for DENV-2 and CHIKV, larger positive Ct offsets indicate later detection by the M10 than by the reference assay at a given viral concentration, consistent with the higher operational LoDs observed for these targets. Conversely, negative Ct offsets for YFV and DENV-1 support the observation that the M10 achieves superior detection for these viruses, consistent with their lower LoDs relative to the reference RT-qPCR (1 vs. 5 PFU/mL for both targets). For DENV-3, the M10 exhibited lower Ct values relative to the RT-qPCR across the tested concentration range, indicating superior amplification efficiency; however, both platforms achieved equivalent operational LoDs of 1 PFU/mL, and therefore the clinical significance of this offset is limited.

The predominance of DENV-3 in this cohort (77.1%) is consistent with PAHO surveillance alerts documenting re-emergence of DENV-3 subtype III across the Americas since 2023, including Central America and Panama [65,70,71]. This resurgence reflects the accumulation of immunologically naïve populations during prolonged low-transmission intervals and underscores the epidemiological value of serotype-resolved platforms [71,72]. The absence of DENV-2 likely reflects local herd immunity and serotype oscillation dynamics during the study period rather than a detection failure, as DENV-2 was not identified by the reference standard either [12,13,73].

Several operational and analytical limitations should be acknowledged. The most substantive limitation of this study is the restriction of clinical diagnostic evaluation to dengue. Clinical specimens from patients with confirmed ZIKV, CHIKV, YFV, or WNV infection were not available in the study setting, reflecting the epidemiological dominance of dengue among acute febrile illness presentations in Panama during the study period and the operational constraints of retrospective sample collection in decentralized health facilities. Consequently, sensitivity and specificity for these non-dengue targets cannot be inferred from the present data. Phase 2 was incorporated specifically to address this gap by characterizing analytical detection thresholds across all panel targets under controlled conditions; however, analytical sensitivity data derived from cultured viral stocks do not substitute for clinical validation in patient specimens, where viral load kinetics, sample matrix effects, and host factors may influence assay performance. Prospective studies enrolling patients with confirmed non-dengue arboviral infection are therefore required to complete the clinical performance profile of the panel.

Reliable RT-qPCR performance is contingent on specimen integrity [74,75,76,77]; pre-analytical conditions account for a substantial proportion of total errors in molecular laboratory practice and can reduce RT-qPCR sensitivity even when NS1 detection is preserved [74,76]. Although strict exclusion criteria and verified storage conditions were applied, residual pre-analytical variability cannot be entirely excluded. Prompt serum separation, validated cold-chain maintenance, and minimal freeze–thaw cycles therefore remain indispensable prerequisites for reliable multiplex RT-qPCR implementation in both clinical and surveillance settings [39,74,76,77,78,79].

Additionally, positive YFV signals in individuals with recent 17D vaccine immunization may be indistinguishable from wild-type infection by multiplex RT-PCR, necessitating interpretation in conjunction with vaccination history and clinical–epidemiological data [80]. While routinely achievable in adult patients, the 600 µL cartridge input volume requirement may limit feasibility in pediatric populations. Specifically, obtaining this minimum serum volume for automated processing is often challenging in neonates and young infants [81,82].

These analytical characteristics, including serotype resolution, a sample-to-result time of approximately 60 min, reagent storage at 2–28 °C, and a closed-cartridge format, are operationally relevant in the context of decentralized arboviral testing, where infrastructure and cold-chain constraints limit implementation of conventional open-tube molecular assays [39,51]. Early virological confirmation supports timely clinical triage and informs surveillance of emergent lineages, vaccine deployment decisions, and severity risk stratification [70,83,84]. The platform’s analytical capacity to detect simultaneous arboviral targets in a single reaction, including co-infections relevant to antibody-dependent enhancement, warrants prospective clinical evaluation in cohorts specifically designed to capture co-circulating etiologies [37,46,84,85].

## 5. Conclusions

This study provides an independent two-phase evaluation of the STANDARD M10 Arbovirus Panel in a hyperendemic setting, combining clinical performance estimates for dengue with controlled analytical sensitivity comparisons across eight arboviral targets. In this cohort, the assay showed high agreement with a composite NS1 ELISA-based reference standard for dengue, together with serotype-level resolution and absence of cross-reactivity in the multiplex analytical experiments, supporting its use as a complementary molecular tool for acute dengue diagnosis and arboviral screening rather than as a replacement for existing reference RT-qPCR assays. The two-phase design was adopted to address a recognized operational limitation: the absence of confirmed clinical specimens for non-dengue arboviruses in the study setting. Phase 2 therefore serves as a controlled analytical complement to the clinical phase, providing reference data on detection thresholds across all eight panel targets rather than clinical performance estimates. The reduced analytical sensitivity observed for DENV-2 and CHIKV highlights that panel-wide performance is target dependent and should be interpreted cautiously, particularly at low viral loads. Further prospective, multicenter studies that include clinical specimens positive for ZIKV, CHIKV, YFV and WNV, along with formal LoD_95_ determinations, are needed to complete the performance profile of the panel and to robustly define its role within comprehensive arboviral syndromic surveillance strategies in the Americas.

Dengue, Zika, Chikungunya, Yellow fever, and West Nile viruses co-circulate across the Americas and cause overlapping clinical syndromes during the acute phase of infection. This study evaluated the STANDARD M10 Arbovirus Panel in Panama using a two-phase design. In the clinical phase, the assay demonstrated high agreement with the reference standard for dengue detection and provided serotype-level information, with DENV-3 predominating. In the analytical phase, operational limits of detection were comparable to reference RT-qPCR for six of eight targets; however, substantially reduced analytical sensitivity was observed for DENV-2 and Chikungunya virus. Because confirmed clinical specimens for ZIKV, CHIKV, YFV, and WNV were not available in the study setting, a controlled analytical phase was incorporated to characterize detection limits across all eight panel targets using characterized reference strains. These findings support the use of the M10 as a complementary molecular tool for dengue diagnosis and multiplex arboviral screening; prospective clinical validation of non-dengue targets remains necessary to complete the performance profile of the panel.

## Figures and Tables

**Table 1:** Clinical performance of the STANDARD M10 Arbovirus Panel for dengue detection against the composite reference standard (NS1 ELISA). The analysis included 163 serum samples: 100 classified as dengue reference-positive and 63 as reference-negative. The M10 correctly identified 96 of 100 positive samples and 63 of 63 negative samples (0 false positives). 95% CIs for sensitivity, specificity, PPV, and NPV were estimated using exact binomial (Clopper–Pearson) methods; CI for Cohen’s κ was estimated using the Fleiss standard error formula.

Metric	Value	95% CI
Sensitivity	96.0% (96/100)	89.8–98.9%
Specificity	100% (63/63)	94.3–100%
PPV	100% (96/96)	96.2–100%
NPV	94.0% (63/67)	85.4–98.3%
Overall accuracy	97.5% (159/163)	93.8–99.3%
Cohen’s κ	0.95	0.90–1.00

## Data Availability

The original contributions presented in this study are included in the article. Further inquiries can be directed to the corresponding authors.

## Supplementary Materials

The following supporting information can be downloaded at: https://www.mdpi.com/article/10.3390/diagnostics16121799/s1, Supplementary Table S1—specificity_summary_table; Supplementary Table S2—Analytical_LoD_Performance_M10_vs_CDC; Supplementary Table S3—STARD 2015 Reporting Checklist; Supplementary Table S4—Regression-based Ct offsets between STANDARD M10 and CDC RT qPCR.

## Abbreviations

The following abbreviations are used in this manuscript:

ATCC	American Type Culture Collection
CBI-HAT	Institutional Research Bioethics Committee of Hospital Aquilino Tejeira
CDC	Centers for Disease Control and Prevention
CHIKV	Chikungunya virus
CI	Confidence interval
CPE	Cytopathic effect
Ct	Cycle threshold
DENV	Dengue virus
ELISA	Enzyme-linked immunosorbent assay
IC	Internal control
ICGES	Gorgas Memorial Institute for Health Studies
IgM	Immunoglobulin M
LoD	Limit of detection
MEM	Minimum Essential Medium
MINSA	Ministerio de Salud de Panamá
NPV	Negative Predictive Value
NS1	Non-structural protein 1
PAHO	Pan American Health Organization
PFU	Plaque-forming unit
PPV	Positive Predictive Value
POCT	Point-of-care testing
R^2^	Coefficient of determination
RT-qPCR	Reverse transcription quantitative polymerase chain reaction
WHO	World Health Organization
WNV	West Nile virus
YFV	Yellow fever virus
ZIKV	Zika virus
