# Supplementary material for: Analytical and Clinical Evaluation of the STANDARD M10 Arbovirus Panel for Dengue Detection, Serotyping, and Multiplex Arboviral Screening in the Americas
Source: Diagnostics (Basel). 2026 Jun 11;16(12):1799. doi: 10.3390/diagnostics16121799 (PMC13297785; doi:10.3390/diagnostics16121799)
Supplement: Supplementary file 1 [file diagnostics-16-01799-s001.zip › Supplementary Table S3 - STARD 2015 Reporting Checklist.pdf]

Supplementary Table S3. STARD 2015 Reporting Checklist

TITLE / ABSTRACT

No.	STARD 2015 Item	Reported	Location in manuscript
1	Identification as a study of diagnostic accuracy using at least one measure of accuracy (sensitivity, specificity, predictive values, or AUC) in the title or abstract	Yes	Title: "Analytical and Clinical Evaluation…"; Abstract Results: sensitivity 96.0%, specificity 100%, PPV, NPV, Cohen's κ — p. 1
2	Structured summary of study design, methods, results, and conclusions	Yes	Abstract — Background, Methods, Results, Conclusions — p. 1

INTRODUCTION

No.	STARD 2015 Item	Reported	Location in manuscript
3	Scientific and clinical background, including the intended use and clinical role of the index test	Yes	Introduction pp. 2–4; intended use stated as complementary molecular POCT for arboviral diagnosis
4	Study objectives and hypotheses	Yes	Introduction, last paragraph p. 4: "the present study evaluated the clinical diagnostic accuracy and analytical performance of the STANDARD M10 Arbovirus Panel…"

METHODS

Study Design

No.	STARD 2015 Item	Reported	Location in manuscript
5	Whether data collection was planned before (prospective) or after (retrospective) the index test and reference standard were performed	Yes	Section 2.1, p. 4: "A **retrospective** analytical study was conducted in two complementary phases."

Participants

No.	STARD 2015 Item	Reported	Location in manuscript
6	Eligibility criteria	Yes	Section 2.2, p. 5: inclusion (clinical suspicion, acute phase 0–5 days, sterile tube, completed data form); exclusion (absent form, volume <1 mL, lipemic/hemolyzed, >5 days onset)
7	On what basis potentially eligible participants were identified	Yes	Section 2.2, p. 5: 101 prospective samples from symptomatic patients (Metropolitan Health Region, Ministry of Health); 62 archived samples from ICGES EQA program
8	Where and when potentially eligible participants were identified (setting, location, dates)	Yes	Section 2.1, pp. 4–5: Universidad de Panama + ICGES, Panama City, Panama; prospective: 2025; archived: ICGES repository
9	Whether participants formed a consecutive, random, or convenience sample	Yes	Section 2.2, p. 5: "Samples were selected by **convenience sampling**. The cohort does not represent a consecutive or randomly sampled diagnostic population."

Test Methods

No.	STARD 2015 Item	Reported	Location in manuscript
10a	Index test in sufficient detail to allow replication	Yes	Section 2.2, p. 6: STANDARD M10 Arbovirus Panel (SD Biosensor); 600 µL serum per cartridge; automated extraction/RT/amplification/detection; 8 targets (DENV-1–4, ZIKV, CHIKV, YFV, WNV); ~60 min; blinded operators
10b	Reference standard in sufficient detail to allow replication	Yes	Section 2.2, p. 6: Panbio Dengue Early ELISA (Abbott); NS1-coated wells; HRP-anti-NS1; TMB; OD 450 nm; cut-offs: negative <9, equivocal 9–11, positive >11 Panbio units [43–45]. Phase 2: CDC RT-qPCR [17, 47–50]; MagMAX™ extraction; iTaq™ One-Step Kit; QuantStudio™ 5
11	Rationale for choosing the reference standard	Yes	Section 2.1, p. 4 and Section 2.2, pp. 5–6: NS1 ELISA + clinical data reflects routine practice in Panama; molecular reference not available for all archived samples; misclassification risk explicitly acknowledged
12a	Definition of and rationale for thresholds to distinguish positive from negative results of the **index test**	Yes	Section 2.2, p. 6: any valid Ct output = positive per manufacturer software ; Section 2.3, p. 9: numeric Ct = positive; "NEGATIVE" output = below operational LoD
12b	Definition of and rationale for thresholds to distinguish positive from negative results of the **reference standard**	Yes	Section 2.2, p. 6: Panbio units manufacturer-defined [43–45]; equivocal results (9–11 units) excluded from accuracy analysis
13a	Whether index test readers were aware of reference standard results	Yes	Section 2.2, p. 6: "All assays were performed by trained personnel **blinded** to the composite reference classification at the time of testing."
13b	Whether reference standard readers were aware of index test results	Yes	Section 2.2, p. 6: composite reference classification was assigned from archived NS1 ELISA results prior to index test analysis; reference classification was not influenced by M10 outcomes
14	Methods for estimating or comparing measures of diagnostic accuracy	Yes	Statistical Analysis, pp. 9–10: sensitivity/specificity/PPV/NPV with 95% CIs by exact binomial (Clopper–Pearson); Cohen's κ (Fleiss SE); linear regression per virus (Phase 2); R^2; $p < 0.05$
15	How indeterminate index test or reference standard results were handled	Yes	Section 2.2, p. 6: equivocal NS1 results (9–11 Panbio units) excluded. No invalid or indeterminate M10 results occurred during Phase 1 testing.
16	How missing data were handled	Yes	Section 2.2, p. 5: 37/200 samples excluded (volume, documentation, quality); no missing core data among 163 included samples
17	Any analyses of variability in diagnostic accuracy, distinguishing pre-specified from exploratory	Yes	Statistical Analysis, pp. 9–10: primary accuracy analysis (sensitivity, specificity, PPV, NPV, κ) pre-specified; DENV serotype distribution and Phase 2 Ct comparisons conducted as secondary/exploratory analyses
18	Intended sample size and how it was determined	Yes	Section 2.2, p. 5: no formal a priori calculation; precision estimate post-hoc — ≥138 positive samples required for sensitivity ≥90% at ±5% margin (95% CI); 100 positives included

RESULTS

No.	STARD 2015 Item	Reported	Location in manuscript
19	Flow of participants, using a diagram	Yes	Section 2.2, p. 5 (narrative): 200 assessed → 37 excluded → 163 analyzed (101 prospective + 62 archived); 100 reference-positive / 63 reference-negative → 96 TP / 4 FN / 63 TN / 0 FP.
20	Baseline demographic and clinical characteristics of participants	Yes	Days from symptom onset, sample source, reference classification for all 163 samples
21a	Distribution of severity of disease in those with the target condition	Partial	Clinical severity classification (WHO 2009) not systematically available for all participants given retrospective design; stated as study limitation in Discussion
21b	Distribution of alternative diagnoses in those without the target condition	Partial	Alternative diagnoses not collected for dengue-negative samples.
22	Time interval and any clinical interventions between index test and reference standard	Yes	Section 2.2, pp. 5–6: prospective samples — NS1 performed as part of routine workup before M10 testing; archived ICGES samples — stored at −80°C (≤1 freeze-thaw); M10 performed after archival; no clinical interventions between original classification and M10 re-testing
23	Cross-tabulation of index test results by reference standard results	Yes	Table 1, p. 11: TP = 96, FP = 0, FN = 4, TN = 63 (n = 163); no indeterminate or missing results
24	Estimates of diagnostic accuracy and their precision (95% CIs)	Yes	Table 1, p. 11: Sensitivity 96.0% (89.8–98.9%); Specificity 100% (94.3–100%); PPV 100% (96.2–100%); NPV 94.0% (85.4–98.3%); Accuracy 97.5% (93.8–99.3%); κ = 0.95 (0.90–1.00)
25	Any adverse events from performing the index test or reference standard	N/A	Study used pre-existing de-identified serum samples (retrospective). No adverse events associated with diagnostic procedures.

DISCUSSION

No.	STARD 2015 Item	Reported	Location in manuscript
26	Study limitations, including sources of potential bias, statistical uncertainty, and generalisability	Yes	Discussion pp. 15–19: (1) non-molecular composite reference standard; (2) convenience sampling; (3) no clinical ZIKV/CHIKV/YFV/WNV positives; (4) operational LoD, not formal LoD_{95} (CLSI EP17-A2); (5) residual pre-analytical variability; (6) YFV vaccine strain interpretation caveat
27	Implications for practice, including intended use and clinical role of the index test	Yes	Discussion pp. 18–19; Conclusions p. 19: M10 positioned as complementary decentralized tool for acute dengue diagnosis; not a replacement for reference RT-qPCR; implications for serotype surveillance, clinical triage, and national arboviral monitoring programs

OTHER INFORMATION

No.	STARD 2015 Item	Reported	Location in manuscript
28	Registration number and name of registry	N/A	Study not prospectively registered, consistent with retrospective design using archived and de-identified specimens
29	Where the full study protocol can be accessed	Yes	Full study protocol available from the corresponding author upon reasonable request
30	Sources of funding and other support; role of funders	Yes	Funding section: reagents and financial support for publication costs provided by SD Biosensor, Inc. The manufacturer had no role in study design, data collection, analysis, interpretation, or decision to submit. See also Conflict of Interest disclosure
